# Supplementary material for: Facile and Safe Synthesis of Novel Self-Pored Amine-Functionalized Polystyrene with Nanoscale Bicontinuous Morphology
Source: Int J Mol Sci. 2020 Dec 10;21(24):9404. doi: 10.3390/ijms21249404 (PMC7763285; doi:10.3390/ijms21249404)
Supplement: Supplementary file 1 [file ijms-21-09404-s001.pdf]

Supporting Information

Facile and safe synthesis of novel self-pored amine-functionalized polystyrene with nanoscale bicontinuous morphology

Qilin Gui[a], Qi Ouyang[a], Chunrong Xu[b], Hongxue Ding[a], Shuxian Shi[b], Xiaonong Chen[a]*

[a] Beijing Laboratory of Biomaterials, Beijing University of Chemical Technology, Beijing 100029, China

[b] Key Laboratory of Carbon Fiber and Functional Polymers, Ministry of Education, Beijing University of Chemical Technology, Beijing 100029, China

*Corresponding author. Email: chenxn@mail.buct.edu.cn

Contents

1. Materials 1

2. Instrumentation and Characterization 1

3. Other Characterization 3

1. Materials

N-vinylformamide (NVF) (Aldrich) and styrene (St) (Beijing Chemical Works, Beijing, China) were purified by distillation under reduced pressure. Azobisisobutyronitrile (AIBN) (Tianjin Damao Chemical Reagent Factory, Tianjin, China) was recrystallized. *N,N*-dimethylformamide (DMF), tetrachloride carbon (CCl_4), dimethyl sulfoxide (DMSO), Bisphenol A (BPA), bromoethane and toluene (Beijing Chemical Works, Beijing, China) were used as received.

2. Instrumentation and Characterization

Fourier Transform Infrared Spectroscopy (FTIR). Attenuated total reflection infrared spectroscopy (ATR-FTIR, Nicolet 6700, USA) was used to record the IR spectra at room temperature. Each spectrum was acquired in a wavenumber range from 4000 to 500 cm^{-1} at a resolution of 4 cm^{-1} for 32 scans. Spectra of both homopolymers (i.e. PSt, PNVF) and copolymerized products before and after hydrolysis were recorded.

Nuclear Magnetic Resonance Spectroscopy (NMR). The ^{1}H-NMR measurements were carried out on an AV400 NMR spectrometer (BRUCKER, Switzerland). The samples were dissolved in deuterated dimethylsulfoxide (D-DMSO), and the solutions were measured with tetramethylsilane (TMS) as the internal reference.

Differential Scanning Calorimetry (DSC). Glass transition temperature (T_g) was measured by DSC (DSC822e, METTLER-TOL EDD, Switzerland) in a dry nitrogen atmosphere at a heating rate of 10 °C/min. The samples (about 5-10 mg) were first heated to 150 °C and held at this temperature for 10 min to remove the thermal history, followed by quenching to 20 °C. A heating rate of 10 °C/min was used in all cases. The glass transition temperature (T_g) was taken as the midpoint of the heat capacity change.

Gel Permeation Chromatography (GPC). The molecular weights of the polymer were measured on a Waters HPLC system (WATERS, USA) equipped with a Waters 2410

refractive index detector and a Waters 996 photodiode array detector. A set of monodisperse polystyrene standards were used as calibration standard for GPC. THF was used as an eluent at a flow rate of 1 ml/min at 30 °C.

Scanning electron microscopy (SEM). SEM images of copolymerized products before and after hydrolysis were taken by using a JSM-6700F instrument (JEOL, Japan). The images were obtained at a working distance of ~ 8 mm and at beam voltages of 10 kv.

Atomic force microscope (AFM). AFM (Bruker, USA) was used to observe the bicontinuous structure of the samples. The DMSO solution of NVF-St copolymer (1~3 mg/mL) was dropped on a silicon wafer to form a film by KW4A spin coater (Beijing SETCAS Electronics Co., Ltd) at 3000 r/min for 60s. Samples were annealed at 120 ° C for three hours before testing.

Transmission electron microscope (TEM). TEM (Hitachi, Japan) was used to observe the microphase separation structure of the samples. NVF-St copolymer (2-4 mg/mL) was dissolved in DMSO and the solution was dropped onto a copper grid. Samples were annealed at 120 °C for three hours and dyed with ruthenium tetroxide (RuO_4) for 20 min before testing.

Brunaner-Emmett-Teller analysis (BET). The specific surface area of NVF-St copolymer was measured by Autosorb-iQ (QUANTACHROME, USA) using Brunaner-Emmett-Teller (BET) method. Each sample (20 mg) was degassed at 60 °C for 72 h and then backfilled with N_2.

Ultraviolet-visible analysis (UV-vis). Ultraviolet-visible (UV-vis) spectra were recorded with a UV-Vis 2550 spectrophotometer (SHIMADZU, Japan).

3. Other Characterization

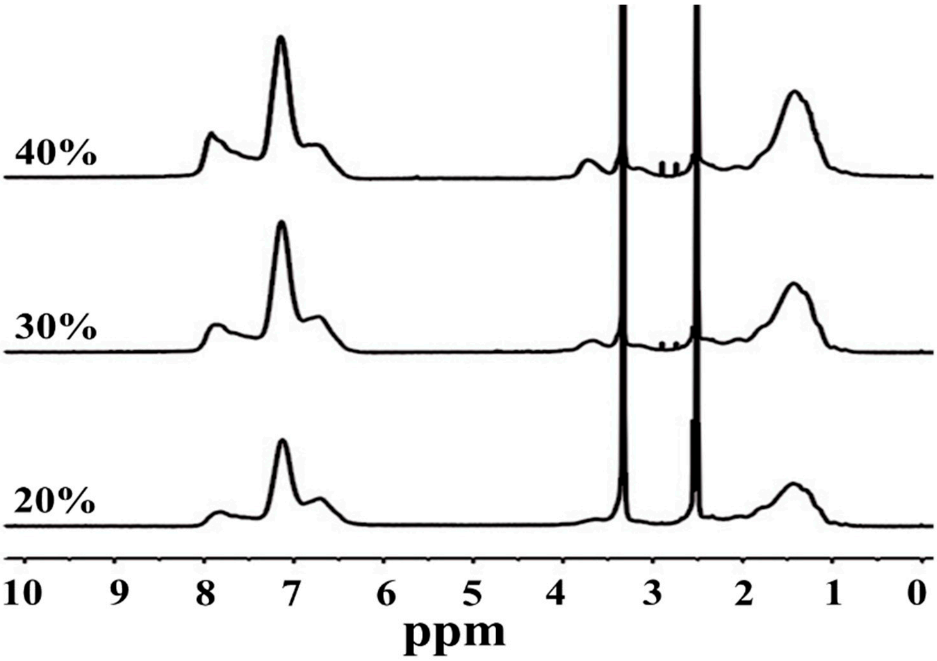

Fig. S1 ^{1}H-NMR spectra of NVF-St copolymers obtained at different monomer concentration (20% No.2, 30% No.5, 40% No.6, molar ratio of NVF/St 83/17).

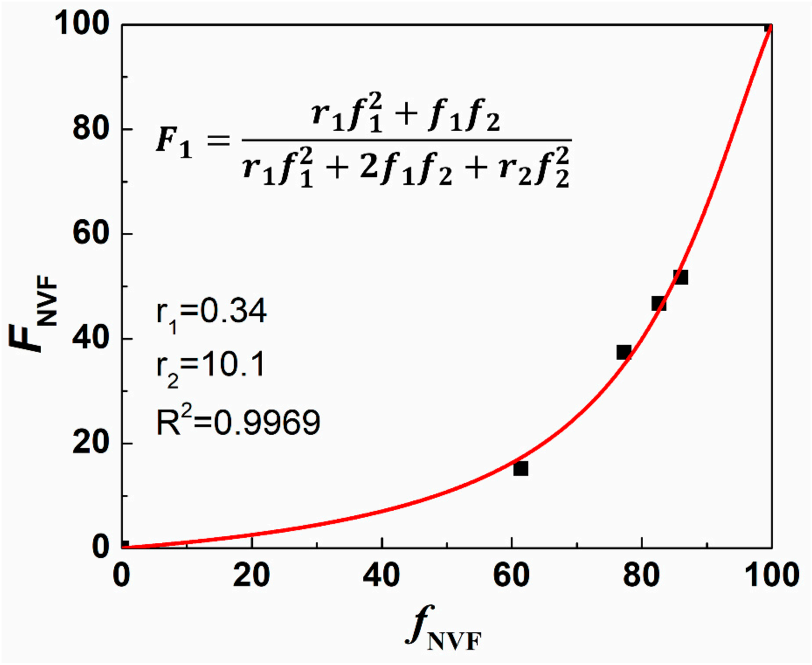

Fig. S2 The relationship between $\boldsymbol{F_{NVF}}$ and $\boldsymbol{f_{NVF}}$ (r_1 NVF, r_2 St).

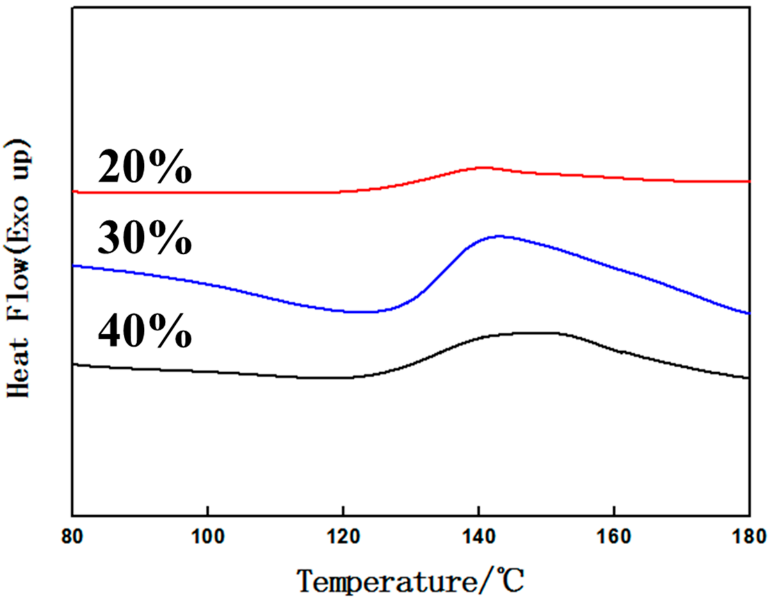

Fig. S3 DSC curves of NVF-St copolymers obtained at different monomer concentration (20% No.2, 30% No.5, 40% No.6, molar ratio of NVF/St 83/17).

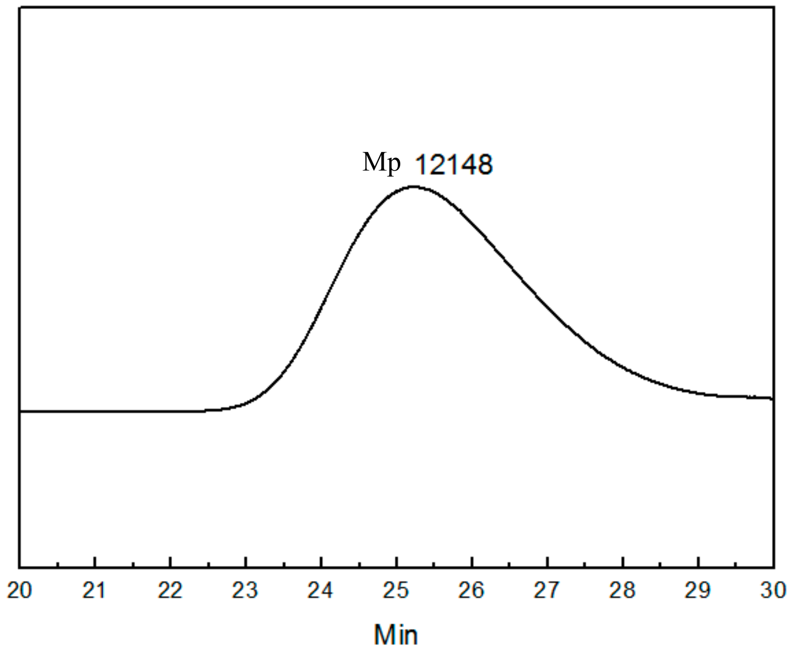

Fig. S4 GPC curve of the NVF-St copolymer (No.1 in Tab. 1)

Tab. S1 Specific surface area of the copolymer sample obtained through different drying conditions*

Sample	Weight/mg	Condition	specific surface area/m^2g^{-1}
**No.6**	20.3	Vacuum-dried	3.5
**No.6**	20.3	Freeze-dried	80.5

*No.6 in Tab. 1, molar ratio of NVF/St 83/17, NVF mass percentage 58.0%, sample weight 20.3 mg.
